# Model Thermohaline Trends in the Mediterranean Sea during the Last Years: A Change with Respect to the Last Decades?

**DOI:** 10.1100/2012/365698

**Published:** 2012-04-30

**Authors:** F. Javier Soto-Navarro, Francisco Criado-Aldeanueva

**Affiliations:** Physical Oceanography Group, Department of Applied Physics II, University of Málaga, 29071 Málaga, Spain

## Abstract

Temperature and salinity outputs from ECCO (years 93–09) and GLORYS (years 03–09) models have been used to compute the thermohaline and steric sea level trends in the surface (0–150 m), intermediate (150 m–600 m), and deep (600 m–bottom) layers of the Mediterranean Sea. Some changes with respect to the second half of the 20th century have been observed: the cooling of the upper waters of the entire eastern basin since 1950 seems to have vanished; the warming of WMDW historically reported for the second half of the last century could have reversed, although there is no agreement between both models at this point (trends of different sign are predicted); the salinification of WMDW reported for the previous decades is not observed in the south-westernmost area in the period 93–09, and a clear change from positive to negative in the steric sea level trend with respect to the period 93–05 is detected due to the sharp decreasing steric sea level of years 02–06.

## 1. Introduction

**Figure 1:**
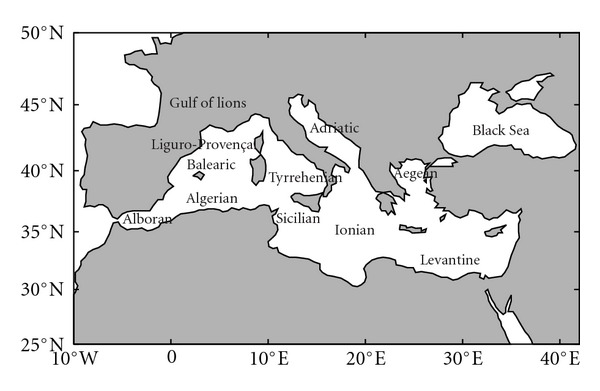
Map of the Mediterranean Sea. The main basins and subbasins are indicated.

The Mediterranean Sea (Figure 1), a semienclosed basin that extends over 3000 km in longitude and over 1500 km in latitude with an area of 2.5·10^12^ m^2^, communicates with the Atlantic Ocean through the Strait of Gibraltar and with the Black Sea through the Turkish Bosphorus and Dardanelles Straits. The Sicily Channel separates the western and eastern Mediterranean basins. The circulation of the Mediterranean Sea is usually described in a schematic way as an open thermohaline cell with two closed secondary cells, one for each subbasin [1]. The principal cell describes the transformation of the Surface Atlantic Water (SAW) to the Levantine Intermediate Water (LIW), which is the main contributor to the Mediterranean outflow into the Atlantic. The two secondary cells describe the transformation of surface and intermediate water to Western Mediterranean Deep Water (WMDW) in the Gulf of Lions and to Eastern Mediterranean Deep Water (EMDW) mainly in the south Adriatic. The sill in the Strait of Sicily prevents a direct communication between the EMDW and WMDW but coupling is achieved via the LIW layer.

Over the past few decades, a series of important changes such as the increase in the temperature and salinity of the WMDW [2, 3], the appearance of a deep water formation site in the southern Aegean Sea [4] and some changes in the LIW characteristics both in the eastern and western basins [5, 6] have been taking place. Since all water masses are closely related and any significant modification involving a single water mass may propagate its effect to the others, thermohaline trends of Mediterranean waters are nowadays a topic of interest as it can be used for predicting correlated changes and diagnose climatic trends.

Several works have dealt with the evaluation of the temperature and salinity trends during the second half of the 20th century in the Mediterranean Sea or specific subbasins using the experimental data available and/or numerical simulations [3, 7–17]. Results differ depending on the analyzed areas and time periods but most of them coincide in positive temperature trends for the last decades in the surface and deeper layers, while the intermediate layer does not show statistically significant trends (i.e., 0.15·10^−2°^C/year (in situ data) to 0.44·10^−2°^C/year (model data) in the basin-averaged 600 m–bottom layer for the period 1965–1998 in [17]). For salinity, the estimations agree in positive trends, with slightly different values in all layers (i.e., 0.06·10^−2^ year^−1^, 0.11·10^−2^ year^−1^, and 0.05·10^−2^ year^−1^ for the basin-averaged surface, intermediate and deep layers, respectively, for 1965–1998 and higher values in the western Mediterranean, see [16, 17] and references herein).

The above-mentioned results are referred to periods previous to year 2002, when the MEDAR/MEDATLAS dataset (MEDAR, 2002) finishes, but recent works show a higher salinity and temperature increase in the Atlantic waters inflowing through the Strait of Gibraltar in the last decade [18, 19] that may be reflected in the Mediterranean. The present work aims at the estimation of these trends for the last two decades using data from two different model simulations. The paper is organised as follows: Section 2 describes the data and methodology; in Section 3 the main results are presented and discussed both for temperature, salinity, and steric sea level trends. Finally, Section 4 summarises the conclusions.

## 2. Data and Methods

We have compared the temperature and salinity trends estimated from the output data of two different simulations: the Jet Propulsion Laboratory Estimating the Circulation and Climate of the Ocean (ECCO model) and the Global Ocean Reanalyses and Simulations (GLORYS1-V1) from the Mercator Ocean group.

The ECCO model data have 1°  ×  1° spatial resolution, 46 depth levels with 10 meter interval for the first 150 meters and a temporal resolution of 10 days. The simulation uses the NCEP (National Centers for Environmental Prediction)/COADS (Comprehensive Ocean-Atmosphere Data Sets) reanalyses as forcing (see [20] for details). The GLORYS dataset has 1/4°  ×  1/4° spatial resolution and uses 50 vertical levels with a finer spacing near the surface (1 m) increasing with depth. It uses different reanalysis products as forcing: the European Space Agency remote sensing satellite (ERS) for the wind, NCEP/NCAR reanalysis for the heat fluxes and evaporation (derived from latent heat flux), and CMAP for precipitation, humidity, and cloud cover (see [21] for details). The covered period is January 2002–December 2008, and the originally daily data have been weekly averaged for this study.

Vertically integrated time series of salinity and temperature have been constructed for three different layers representative of the major water masses in the Mediterranean [11, 22]: the upper layer, 0–150 m, dominated by the inflow of Surface Atlantic Waters (SAW), the intermediate layer, 150–600 m, occupied by the salty and warm Levantine Intermediate Water (LIW) and the deeper layer, 600 m–bottom, filled by the colder and denser Eastern/Western Mediterranean Deep Waters (E/WMDW). To focus on interannual variability and trends, the seasonal signal and the mean value have been removed from the time series by subtracting the following function (estimated by least squares fitting):

(1) $$y=a_{0}+A\cos\left(\omega t-\varphi \right),$$

where **ω** is the annual frequency and *a*_0_ the climatological mean value. The linear trends have also been computed by least square fitting and the 95% confidence intervals estimated by a *t*-student test.

## 3. Results and Discussion

### 3.1. Temperature Trends

**Figure 2:**
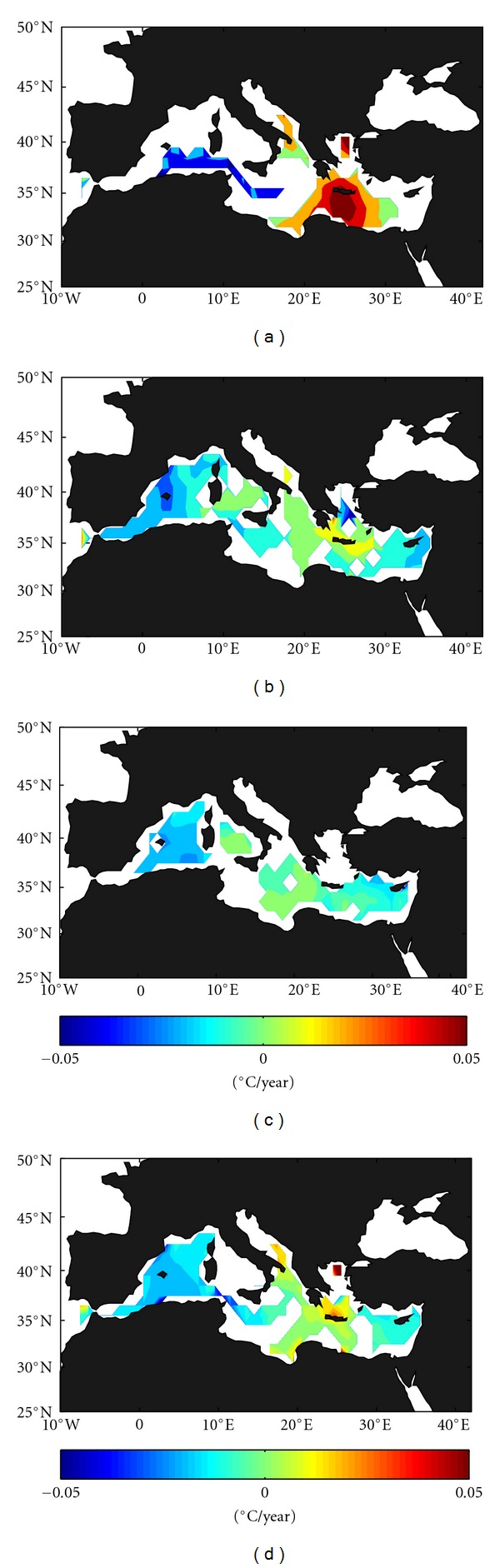
Spatial distribution of the temperature trend from the ECCO model data (93–09) for the different layers: (a) surface layer (0–150 m), (b) intermediate later (150–600 m), (c) deep layer (600 m–bottom), and (d) entire water column.

Temperature trends (from ECCO model, 93–09) in the shallower layer (0–150 m, Figure 2(a)) are not significant (95% confidence interval) in most parts of the Mediterranean. Significant trends are only observed in a small region south of Crete, where a clear positive trend of ~0.05°C/year is found, and along the Algerian coast, with negative values about −0.04°C/year. The cooling of the upper waters of the entire eastern basin reported by Painter and Tsimplis [23] or Vidal-Vijande et al. [17] from MEDATLAS database since 1950 seems to have vanished with the inclusion of the recent most years. In the intermediate layer (150 m–600 m, Figure 2(b)), negative trends are dominant almost everywhere, especially in the western Mediterranean (with values between −0.02°C/year and −0.03°C/year) and in the eastern Levantine subbasin (−0.01°C/year to −0.02°C/year). Positive trends of ~0.01°C/year are only found around Crete, whereas the central Mediterranean is of practically neutral trend. In the deeper layer (600 m–bottom, Figure 2(c)), negative trends are again dominant in the eastern Levantine subbasin with values of −0.01°C/year and in the Algeric-Balearic subbasin (about −0.02°C/year), with this suggesting that the warming of WMDW historically reported [2, 11, 16, 17] especially for the second half of the 20th century could have been reversed. The spatial pattern of the depth-averaged water column trends (Figure 2(d)) closely follows that of the intermediate and deeper layers, with negative trends in the easternmost and westernmost subbasins (ranging from −0.01°C/year to −0.03°C/year), a reduced region of positive (up to 0.02°C/year) trend around Crete and neutral or nonsignificant trends in the central Mediterranean. This last result is in reasonably good agreement with Criado-Aldeanueva et al. [13], who perform a similar analysis (only for the depth-averaged water column) from ECCO data for 1993–2005.

**Figure 3:**
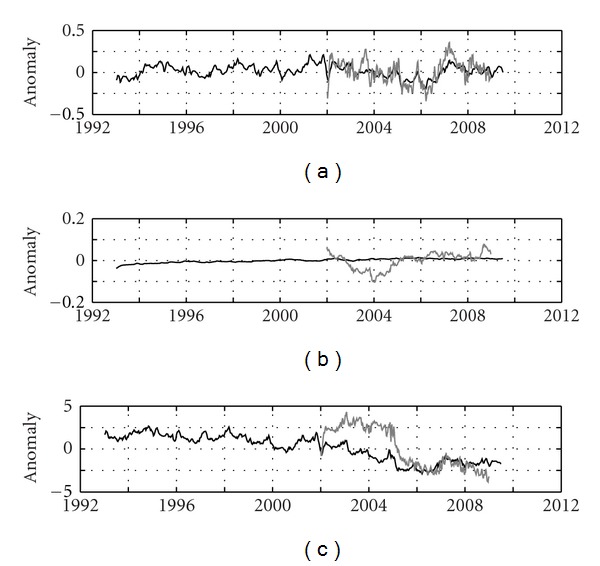
Mediterranean-averaged temperature panel (a), salinity panel (b), and steric sea level panel (c) anomalies for the entire water column from ECCO (black line) and GLORYS (gray line) model data.

The Mediterranean-averaged temperature anomalies for the entire water column are displayed in Figure 3(a), and computed trends for each layer are summarised in Table 1. For the whole period, 93–09 (ECCO model), trends are only significant in the deeper layer (−8.00 ± 0.09*·*10^-3°^C/year) and in the surface layer (positive, although subject to high uncertainty). For comparison purposes, GLORYS data have been superposed (gray line) to ECCO data (black line). A visual good agreement, also reflected in the computed trends, is observed for the common period and this is also the case for the surface and intermediate layers (not shown). Although the trend for the period 02–09 in the surface layer is subject to large uncertainty (see Table 1), two intervals of different trend can be identified: from 02 to 06, a negative trend of −0.10 ± 0.02°C/year and −0.12 ± 0.02°C/year for ECCO and GLORYS, respectively, is observed, followed by a positive trend of 0.16 ± 0.03°C/year (ECCO) and 0.15 ± 0.04°C/year (GLORYS) in 06–09. The same as above is applicable for the entire water column with negative trends of −0.039 ± 0.007°C/year (ECCO) and −0.05 ± 0.01°C/year (GLORYS) in 02–06 that change to positive 0.05 ± 0.01°C/year (the same for both models) in 06–09. Criado-Aldeanueva et al. [13] suggested the possibility of a trend change from 2001, but, with longer datasets available, it seems more likely related to interannual to interdecadal variability rather than to a trend itself. The same trend is observed (−17 ± 2·10^−3°^C/year) for the intermediate layer, this pointing to a cooling of LIW in the last decade, but, surprisingly, great discrepancy is found in the deeper layer, where both models predict trend of different sign (negative for ECCO and positive for GLORYS, that could match the historical warming trends). We lack of a definite explanation for this fact that might be related to the different parameterisation of each model and the resolution of the atmospheric forcing that drive deep-water formation processes but, in any case, longer datasets are necessary to check this behaviour.

### 3.2. Salinity Trends

**Figure 4:**
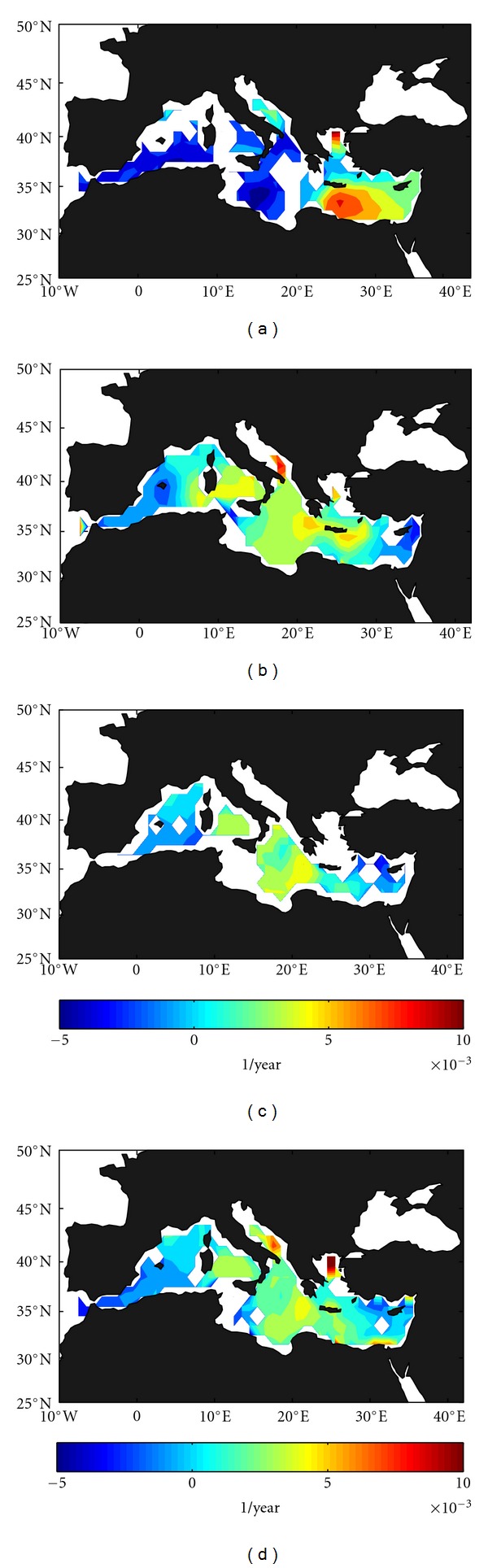
Spatial distribution of the salinity trend from the ECCO model data (93–09) for the different layers: (a) surface layer (0–150 m), (b) intermediate later (150–600 m), (c) deep layer (600 m–bottom), and (d) entire water column.

Salinity trends (from ECCO model, 93–09) in the shallower layer (0–150 m, Figure 4(a)) are negative in the Ionian basin (up to −5·10^−3^ year^−1^) and in the Alboran Sea and southern Balearic basin, whereas positive values concentrate in the Levantine basin, especially south of Crete with values up to 10·10^−3^ year^−1^. It is interesting to notice the spot of high salinity (and also temperature, see Figure 2(a)) positive trend observed in this layer in the northern Aegean, that may be related to natural or anthropogenic actuations in this area (i.e., on the Maritsa river). Higher resolution studies, out of the scope of this work, are necessary to confirm this hypothesis. In the intermediate layer (150 m–600 m, Figure 4(b)), positive trends (up to 5·10^−3^ year^−1^) are dominant almost everywhere, especially in the central Mediterranean (from Sardinia to Crete) and negative trends are reduced to the eastern Levantine and the westernmost Alboran and Balearic subbasins (−2 to −3·10^−3^ year^−1^). This salinification of LIW extends back to the second half of the 20th century [3, 11, 16, 17] and could have been influenced by internal redistribution of salt in connection with the Eastern Mediterranean Transient in the last decades [4, 24–26]. In the deeper layer (600 m–bottom, Figure 4(c)), this spatial pattern remains, although the trend becomes nonsignificant in more areas and the salinification of WMDW reported for the previous decades [2, 11, 16, 17] is not observed in the south-westernmost area. This is consistent with the results for temperature commented above and could reinforce the hypothesis of a trend change of the WMDW in the last years. Again, the spatial pattern of the depth-averaged water column trends (Figure 4(d)) closely follows that of the intermediate and deeper layers, with negative trends in the Alboran and western Balearic subbasins (~−2·10^−3^ year^−1^) and positive trends almost everywhere with higher values (up to 5·10^−3^ year^−1^) in the Tyrrhenian and southern Ionian and Aegean. Compared with Criado-Aldeanueva et al. [13], who perform a similar analysis (only for the depth-averaged water column) from ECCO data for 1993–2005, salinity trend has increased in the Tyrrhenian and southern Ionian, due to the effect of the last years included in the time series.

Figure 3(b) shows the Mediterranean-averaged salinity anomalies for the entire water column, and Table 1 summarises the computed trends for each layer, all of them positive and significant at 95% confidence level. Focusing on ECCO model, the computed trends for the whole period 93–09 are rather similar to those for the last decade 02–09 in the surface and deeper layer (~1.3·10^−3^ year^−1^ and ~1.1·10^−3^ year^−1^, resp.). But in the intermediate layer, the trend for the last decade is about three times lower than for the entire period, this suggesting some attenuation in the salinity increase in the last years. When comparing with GLORYS data (gray line), some discrepancies are observed, especially in the period 02–05, where GLORYS anomalies are much noisier than those of ECCO due to the effect of the surface layer (not shown) since the intermediate and deeper layers (not shown) are much smoother. Again the different parameterisation (i.e., the sea surface salinity restoring term that affects the interannual variability) and resolution of both models along with the climatological fields used for the forcing could be invoked to explain the discrepancies. For the common period 02–09, GLORYS trends are higher than those of ECCO in all layers, especially in the deeper one (9·10^−3^ year^−1^ versus 1.18·10^−3^ year^−1^). Although both models point to a positive trend in all layers, it is necessary to refine these estimations to more precisely define its value. GLORYS estimations seem to better reproduce recent experimental studies that find positive trends of that order in the Atlantic waters entering the Mediterranean through the Strait of Gibraltar [18, 19]. However, more exhaustive comparisons between experimental and model results are necessary to state.

### 3.3. Steric Sea Level Trends

Changes in the steric contribution (*ξ*_*S*_) of sea level represent the effect of expansion and contraction of water column due to changes in density (*ρ*) and can be computed from temperature and salinity fields according to

(2) $$\begin{array}{cc} \xi_{S} & =-\frac{1}{\rho_{0}}\overset{0}{\underset{-H}{\int }}{\frac{\partial \rho \left(S,T,P\right)}{\partial T}|}_{T,P=cte}\cdot T^{\prime }\left(z\right)dz\\ & \;+\frac{1}{\rho_{0}}\overset{0}{\underset{-H}{\int }}{\frac{\partial \rho \left(S,T,P\right)}{\partial S}|}_{S,P=cte}\cdot S^{\prime }\left(z\right)dz, \end{array}$$

where *T*′(*z*) and *S*′(*z*) are temperature and salinity anomalies referred to their climatic mean value; *ρ*_0_ represents a reference density, and *H* is the bottom depth. Increases (decreases) of temperature and/or decreases (increases) of salinity lead to positive (negative) *ξ*_*S*_  anomaly.

**Figure 5:**
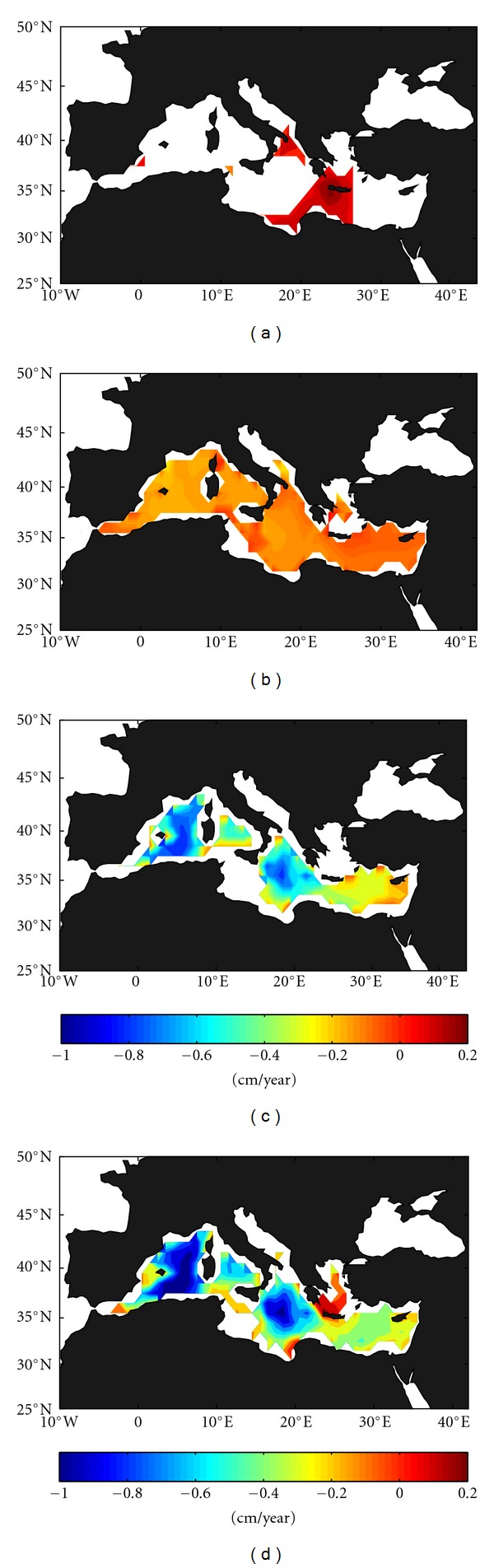
Spatial distribution of the steric sea level trend from the ECCO model data (93–09) for the different layers: (a) surface layer (0–150 m), (b) intermediate later (150–600 m), (c) deep layer (600 m–bottom), and (d) entire water column.

Steric sea level trends (from ECCO model, 93–09) in the shallower layer (0–150 m, Figure 5(a)) are only significant (95% confidence level) in a small region around Crete, where a clear positive trend of 0.1 to 0.2 cm/year is found. The thermosteric effect seems to be dominant in this region since a positive temperature trend (see Figure 2(a)) drives a positive steric trend despite the negative halosteric contribution (see Figure 4(a)), as previously mentioned by Tsimplis and Rixen [10]. In the intermediate and deeper layers (Figures 5(b) and 5(c)), negative trends are found everywhere with higher values (about −0.2 cm/year in the intermediate layer and up to −0.8 cm/year in the deeper layer) in the Balearic subbasin driven by the temperature negative trend (Figures 2(b) and 2(c)). In the central Mediterranean, where temperature trends are neutral or nonsignificant, the salinity increase leads to a negative steric trend on halosteric origin (compare, for instance, Figures 4(c) and 5(c)). In the Levantine subbasin, lower trends are observed (~−0.1 cm/year in the intermediate layer to ~−0.3 cm/year in the deeper layer) due to thermal and haline contributions of opposite sign. For the entire water column (Figure 5(d)), the spatial pattern closely mirrors that of these two layers. The increase in the depth-averaged water column salinity trend in the Tyrrhenian and southern Ionian with respect to the period 1993–2005 [13] leads to a higher negative trend in these areas than obtained by these authors.

The Mediterranean-averaged steric sea level anomalies for the entire water column are displayed in Figure 3(c), and computed trends for each layer are summarised in Table 1. For the whole period 93–09 (hence ECCO model), trends are negative in all layers (except in the surface layer where the fitting is non-significant) due to the concomitant effect of average cooling and salinification of waters. For the entire water column, a negative trend of −0.28 ± 0.01 cm/year is found, which means a clear change from the 0.11 ± 0.03 cm/year obtained by Criado-Aldeanueva et al. [13] for the period 93–05. The years 02–06 are of sharp decreasing steric sea level, as previously shown by Vigo et al. [27], and this has made trend reverse. In any case, trends are sensitive to the length of the series analysed: the longer the series, the more reliable the trend obtained. Since the thermosteric effect seems to dominate, a relative good agreement is found between ECCO and GLORYS series for the common period (Figure 3(c)) although the great salinity discrepancy in 02–05 also reflects here and a more negative trend of −1.01 ± 0.06 cm/year is observed from GLORYS in 03–09 for the entire water column. Longer datasets will be of great help to establish a more reliable comparison.

## 4. Summary and Conclusions

We have compared the temperature, salinity, and steric sea level trends estimated from the output data of two different simulations: ECCO (period 93–09) and GLORYS (period 03–09) models. In general, a reasonable good agreement is found between them, although some discrepancy in salinity in 02–05 (that also reflects in the steric sea level) and temperature trend of different sign in the deep layer are worth mentioning. Some changes with respect to the second half of the 20th century have been observed: (i) the cooling of the upper waters of the entire eastern basin reported by Painter and Tsimplis [23] or Vidal-Vijande et al. [17] from MEDATLAS database since 1950 seems to have vanished with the inclusion of the recentmost years; (ii) the warming of WMDW historically reported [2, 11, 16, 17] for the second half of the 20th century could have reversed. However, at this point great discrepancy is found between both models that predict trend of different sign in the deep layer (negative for ECCO and positive for GLORYS, that could match the historical warming trends) so this result must be considered with caution; (iii) the salinification of LIW, which extends back to the 1950s [3, 11, 16, 17], remains in the recentmost years but the trend for the last decade (from ECCO model) is about three times lower than for the entire 93–09 period, with this suggesting some attenuation in the salinity increase in the last years (not evidenced from GLORYS model); (iv) the salinification of WMDW reported for the previous decades [2, 11, 17] is not observed in the south-westernmost area; (v) a negative steric sea level trend of −0.28 ± 0.01 cm/year is found for the entire water column, which means a clear change from the 0.11 ± 0.03 cm/year obtained by Criado-Aldeanueva et al. [13] for the period 93–05. The sharp decreasing steric sea level of years 02–06 has made trend reverse.

## Figures and Tables

**Table 1:** Linear trends of temperature (*T*, °C/year), salinity (*S*, year^−1^) and steric sea level (*ξ*_*S*_, cm/year) for the Mediterranean Sea from ECCO (E), and GLORYS (G) models. The time periods spanned are also indicated.

	*S*_E(93–09)_ · 10^3^	*S*_E(02–09)_ · 10^3^	*S*_G(02–09)_ · 10^3^	*T*_E(93–09)_ · 10^3^	*T*_E(02–09)_ · 10^3^	*T*_G(02–09)_ · 10^3^	*ξ*_SE(93–09)_	*ξ*_SE(02–09)_	*ξ*_SG(02–09)_
0–150	1.4 ± 0.1	1.3 ± 0.4	6 ± 3	5 ± 3	12 ± 12(1)	15 ± 12 (1)	n.s.	n.s.	n.s.
150–600	2.44 ± 0.06	0.68 ± 0.09	4.1 ± 0.2	n.s.	−17 ± 2	−17 ± 2	−0.100 ± 0.003	−0.16 ± 0.07	−0.30 ± 0.02
600-bottom	1.07 ± 0.02	1.18 ± 0.05	9 ± 1	−8.00 ± 0.09	−7.7 ± 0.2	9.3 ± 0.2	−0.321 ± 0.002	−0.346 ± 0.006	−0.67 ± 0.02
0-bottom	1.77 ± 0.07	0.9 ± 0.1	10 ± 1	n.s.	n.s. (2)	n.s. (2)	−0.28 ± 0.01	−0.32 ± 0.04	−1.01 ± 0.06

(1) Although the trend for the entire period 02–09 is subject to large uncertainty, two intervals of different trend can be identified: from 02 to 06, a negative trend of −0.10 ± 0.02°C/year and −0.12 ± 0.02°C/year for ECCO and GLORYS, respectively, is observed, followed by a positive trend of 0.16 ± 0.03°C/year (ECCO) and 0.15 ± 0.04°C/year (GLORYS) in 06–09.

(2) The same as above is applicable for the entire water column with negative trends of −0.039 ± 0.007°C/year (ECCO) and −0.05 ± 0.01°C/year (GLORYS) in 02–06 that change to positive 0.05 ± 0.01°C/year (the same for both models) in 06–09.
